# Editorial: Neuroimaging innovations for encephalitis, neuroinfectious diseases, and neuroinflammation

**DOI:** 10.3389/fneur.2025.1753198

**Published:** 2025-12-12

**Authors:** Hsiuying Wang, Hui Jan Tan, Tracy Fischer

**Affiliations:** 1 Institute of Statistics, National Yang Ming Chiao Tung University, Hsinchu, Taiwan; 2 Department of Medicine, Faculty of Medicine, National University of Malaysia, Kuala Lumpur, Malaysia; 3 Tulane National Biomedical Research Center, School of Medicine, Tulane University, Covington, GA, United States

**Keywords:** neuroinfectious diseases, encephalitis, neuroinflammatory disorders, neuroimaging, electrophysiology

Neuroinfectious diseases, encephalitis, and neuroinflammatory disorders encompass a diverse group of conditions that disrupt the central nervous system (CNS) through infectious, post-infectious, or autoimmune mechanisms (1–3). The substantial overlap in clinical and pathological features often obscures the boundary between infectious and immune-mediated etiologies, highlighting the importance of early recognition and precise diagnostic evaluation. Recent research increasingly applies multimodal strategies, integrating structural and functional neuroimaging with advanced quantitative methods, such as quantitative susceptibility mapping (QSM), deep learning reconstruction–enhanced magnetic resonance imaging (MRI) reconstruction, and computed tomography (CT) assessments, alongside complementary biomarkers, including cerebrospinal fluid (CSF) profiles, autoantibodies, and electroencephalography (EEG) signatures (4–9). These approaches have substantially improved our ability to detect inflammation and neuronal injury, assess disease severity, and develop predictive models and individualized treatment strategies.

This Research Topic highlights the growing role of innovation in neuroimaging, electrophysiology, and biomarker development for understanding neuroinflammatory, neuroinfectious, and autoimmune disorders of the CNS. The included studies illustrate how modern MRI techniques, quantitative EEG analyses, and targeted CSF assessments provide complementary insights that strengthen diagnostic accuracy and advance clinical care.

Several contributions focus on advanced MRI applications and related quantitative imaging modalities. Gkotsoulias et al. demonstrated that deep learning-assisted reconstruction substantially improves QSM image quality in multiple sclerosis (MS), enabling clearer visualization of MS-related biomarkers. Using diffusion tensor imaging along the perivascular space (DTI-ALPS) to examine glymphatic function in patients recovering from COVID-19, He et al. identified dynamic changes associated with cognitive symptoms and fatigue. In addition, Churchill et al. reported persistent white matter microstructural abnormalities linked to emotional health measures in individuals with post-acute COVID-19 syndrome, which may help distinguish post-acute COVID-19 syndrome from symptomatic non-COVID infection. Finally, Essel et al. compared synthetic fluid-attenuated inversion recovery (FLAIR) with conventional FLAIR imaging in MS and found strong agreement in lesion quantification, suggesting that synthetic sequences may serve as a practical alternative for the assessment of MS patients with a low lesion burden. Collectively, these studies highlight the ability of advanced MRI techniques to detect subtle tissue changes that may not be evident on standard imaging.

Other studies highlight the utility of electrophysiological techniques in identifying functional disturbances in the CNS. Liu et al. synthesized quantitative EEG findings associated with psychiatric symptoms after ischemic stroke and identified stage-specific abnormalities in power spectra. In evaluating EEG patterns in antibody-mediated autoimmune encephalitis, Sun et al. reported that EEG abnormalities are common, often more sensitive than MRI, and strongly correlate with disease severity and prognosis. Wu et al. investigated resting-state EEG in convalescent patients with anti-N-methyl-D-aspartate (anti-NMDA) receptor encephalitis and revealed persistent disruptions in rhythmic activity and network connectivity, indicating residual brain dysfunction, despite clinical recovery. Together, these investigations underscore the value of EEG as a non-invasive and sensitive modality for monitoring functional disturbances across a variety of neurological disorders.

Several studies address challenges in diagnosing CNS infections. Tang et al. compared CSF obtained via lumbar puncture vs. external ventricular drainage for the diagnosis of post-operative CNS infections and demonstrated that lumbar puncture provides significantly greater diagnostic sensitivity. Their findings highlight the risk of false-negative results when relying solely on ventricular samples. Ci et al. examined varicella zoster virus (VZV) infection in immunocompetent adults and showed that VZV can involve the CNS in otherwise healthy individuals, typically presenting with mild symptoms and favorable outcomes following antiviral therapy. These findings reinforce the importance of appropriate sampling strategies and thorough clinical evaluation when CNS infection is suspected.

Additional studies focus on autoimmune glial fibrillary acidic protein (GFAP) astrocytopathy and its diverse presentations. Zhao et al. reported three case studies illustrating how GFAP astrocytosis can mimic tuberculous meningitis, autoimmune encephalitis, neuromyelitis optica spectrum disorder, and movement disorders, complicating early diagnosis. Li and Teng characterized area postrema syndrome in GFAP-immunoglobulin G-positive patients and identified imaging and CSF features that help differentiate it from aquaporin-4-positive neuromyelitis optica. Both studies highlight the diagnostic importance of GFAP antibody testing and the benefit of prompt immunotherapy for improving clinical outcomes.

In a study examining seizure manifestations in autoimmune encephalitis, Li et al. reported that seizures frequently occur in the acute phase and are associated with greater initial disease severity, but they do not significantly affect long-term outcomes. Key prognostic indicators included status epilepticus, medical complications, need for respiratory support, disability at discharge, Antibody Prevalence in Epilepsy and Encephalopathy (APE^2^) score, Response to Immunotherapy in Epilepsy and Encephalopathy (RITE^2^) score, intensive care requirements, and albumin levels. These findings assist clinicians in identifying high-risk patients who may require intensified monitoring during early disease stages.

In summary, this collection of 12 articles illustrates how advances in neuroimaging, electrophysiology, and CSF analysis are transforming our understanding of neuroinflammation, neuroinfection, and autoimmune encephalitis. Together, these studies demonstrate the value of multimodal assessment for improving diagnostic precision, clarifying disease mechanisms, and informing clinical decision-making. We hope this Research Topic contributes meaningfully to the ongoing evolution of neuroimaging innovations and biomarker-driven approaches for the study and management of CNS disorders.
